# HPV types, HIV and invasive cervical carcinoma risk in Kampala, Uganda: a case-control study

**DOI:** 10.1186/1750-9378-6-8

**Published:** 2011-06-25

**Authors:** Michael Odida, Sven Sandin, Florence Mirembe, Bernhard Kleter, Wim Quint, Elisabete Weiderpass

**Affiliations:** 1 Department of Medical Epidemiology and Biostatistics, Karolinska Institutet, Box 281, 171 77 Stockholm, Sweden; 2 Department of Pathology, Faculty of Medicine, Makerere University, P.O. Box 7072, Kampala, Uganda; 3 Department of Gynecology and Obstetrics, Faculty of Medicine, Makerere University, P.O. Box 7072, Kampala, Uganda; 4 DDL Diagnostic Laboratory, Fonteynenburghlaan 7, 2275 CX Voorburg, the Netherlands; 5 Department of Etiological Research, Cancer Registry of Norway, PB 5313 Majorstuen, 0304 Oslo, Norway; 6 Department of Community Medicine, University of Tromsø, N-9037 Tromsø, Norway; 7 Department of Genetic Epidemiology, Folkhälsan Research Center, Biomedicum Helsinki, PB 63, FI-00014 HU, Finland

## Abstract

**Background:**

While the association of human papillomavirus (HPV) with cervical cancer is well established, the influence of HIV on the risk of this disease in sub-Saharan Africa remains unclear. To assess the risk of invasive cervical carcinoma (ICC) associated with HIV and HPV types, a hospital-based case-control study was performed between September 2004 and December 2006 in Kampala, Uganda. Incident cases of histologically-confirmed ICC (N=316) and control women (N=314), who were visitors or care-takers of ICC cases in the hospital, were recruited. Blood samples were obtained for HIV serology and CD4 count, as well as cervical samples for HPV testing. HPV DNA detection and genotyping was performed using the SPF_10_/DEIA/LiPA_25_ technique which detects all mucosal HPV types by DEIA and identifies 25 HPV genotypes by LiPA version 1. Samples that tested positive but could not be genotyped were designated HPVX. Odds ratios (OR) and 95% confidence intervals (CI) were calculated by logistic regression, adjusting for possible confounding factors.

**Results:**

For both squamous cell carcinoma (SCC) and adenocarcinoma of the cervix, statistically significantly increased ORs were found among women infected with HPV, in particular single HPV infections, infections with HPV16-related types and high-risk HPV types, in particular HPV16, 18 and 45. For other HPV types the ORs for both SCC and adenocarcinoma were not statistically significantly elevated. HIV infection and CD4 count were not associated with SCC or adenocarcinoma risk in our study population. Among women infected with high-risk HPV types, no association between HIV and SCC emerged. However, an inverse association with adenocarcinoma was observed, while decrease in CD4 count was not associated with ICC risk.

**Conclusions:**

The ORs for SCC and adenocarcinoma were increased in women infected with HPV, in particular single HPV infections, infections with HPV16- and 18-related types, and high-risk HPV types, specifically HPV16, 18 and 45. HIV infection and CD4 count were not associated with SCC or adenocarcinoma risk, but among women infected with high-risk HPV types there was an inverse association between HIV infection and adenocarcinoma risk. These results suggest that HIV and CD4 count may have no role in the progression of cervical cancer.

## Background

Human papillomavirus (HPV) infection is a necessary cause for the development of cervical cancer, and the risk of cervical cancer differs [1] according to HPV type. HIV infection has also been associated with cervical cancer risk [2], which seems to vary according to co-infection with different HPV types [3,4]. The association between HIV and cervical cancer appears to be less evident in low-income countries, notably sub-Saharan Africa, than in high-income countries [5,6]. Two studies conducted at the beginning of the AIDS epidemic in Uganda [7] and Tanzania [8], which assessed HIV infection and cervical cancer, showed no association. However, a positive association has been observed in Western countries such as Italy, France and Spain [5,9,10], as well as in some recent studies from Uganda [11] and Tanzania [12]. Whether these differences in cervical cancer risk between studies can be explained by differences in co-infection with specific HPV types remains unclear.

We present here results from a study of invasive cervical carcinoma (ICC) in relation to HPV status, HIV status and CD4 count in Uganda, where the estimated world-standardised incidence rates of cervical cancer is rising and is now 52.4 per 100 000 women [13].

## Methods

We conducted a hospital-based case-control study in Mulago Hospital in Kampala, Uganda, which is the national referral and teaching hospital for Makerere University. The hospital admits about 30 ICC cases each month.

Patients attending the gynaecological clinics or emergency section are mostly residents of Kampala City and the surrounding areas, with a population of about 1 000 000, although some come directly from areas outside Kampala. Like most urban areas of Uganda, HIV prevalence is relatively high in Kampala [14]. Recruitment of ICC cases and control women was done by selected nurses and midwives working in the gynaecological wards or clinics, using the inclusion and exclusion criteria described below.

### Recruitment of ICC cases

Patients eligible for the study were women aged 18 to 74 years, residents of Uganda for at least 2 years and consecutively diagnosed with incident ICC during the period September 2004 to September 2006. ICC cases recruited had not yet undergone primary treatment, signed a written informed consent form to participate in the study, and were able to provide biological samples. ICC cases were excluded if tissue and blood samples could not be collected because they were in a terminal stage, or for any other reason that might interfere with established patterns of patient care.

### Recruitment of control women

At the Mulago Hospital, all hospitalised patients have one or more accompanying persons, who are responsible for preparing food and taking care of basic hygiene needs and other patient requirements. These accompanying persons are in general female members of the same family or clan. Control women were recruited among those accompanying or visiting ICC cases. The motivation to enrol relatives of the cancer patients as controls was to avoid bias by including control women who would most likely live in the same areas of the country as the ICC cases and would have a similar social background. In addition, control women would probably have used the same hospital as the ICC cases if they had had a cervical cancer diagnosis. Since the evaluation of family history of cervical cancer was not part of our study hypothesis, the inclusion of blood relatives of ICC cases did not constitute a problem.

Control women were frequency matched to ICC cases by 5-year age groups. Inclusion criteria for control women were the same as for ICC cases (i.e. resident of Uganda for at least 2 years, aged 18 to 74 years between September 2004 and December 2006, able to give informed consent and willing to provide biological samples), save the cervical cancer diagnosis.

Control women were offered a pelvic examination with visual inspection of the cervix uteri and Pap smear screening test. Test results were made available to control women before they left the hospital (i.e. during the period of hospitalisation of the ICC cases they accompanied). Women in whom pre-malignant and malignant cervical abnormalities were suspected or detected were referred for standard follow-up diagnosis and treatment at Mulago Hospital. A symptomatic diagnosis of cervical and vaginal infections was done by the gynaecologists examining control women, and appropriate antibiotic treatment was offered free of charge whenever indicated.

### Collection of biological samples and study procedures

Information about reproductive history, lifestyle practices and sexual behaviour was assessed using a standardised questionnaire administered by the nurses or midwives recruiting ICC cases and control women. Blood was obtained for a full blood count, HIV serological testing, and CD4 count. All subjects underwent pelvic and abdominal examinations, and cervical samples were obtained for diagnosis and HPV detection.

For ICC cases, biopsies of the cervical lesions were collected under general anaesthesia. Samples were immediately stored in sterile specimen tubes and transported in a flask containing ice to the pathology laboratory. There the specimen was divided into two parts: one part was fixed in 10% buffered formalin (for 12 to 24 hours) for histological processing and diagnosis, and the other half was kept in a Nunc tube, labelled and stored at -70°C until shipped for HPV testing. Biopsy samples were processed using the automatic tissue processor; they were paraffin embedded, sectioned at 4 μm thickness and stained with Haematoxylin and Eosin.

For control women, exfoliated cervical cells were collected with two Cervex brushes before any treatment for cervical or vaginal infection was administered. The first brush was rinsed in PreservCyt solution (ThinPrep, Hologic, Marlborough, MA, USA) according to the manufacturer's instructions and kept at room temperature until shipped to the laboratory for HPV testing. The second brush was used to make a smear and stained with Pap stain; smears were classified as normal or abnormal. All histological and cytological diagnoses were done by a pathologist at the Department of Pathology, Makerere University.

### HIV testing and CD4 count

HIV was measured using a rapid test [15], which was the recommended test in Uganda during the study period (Ministry of Health of Uganda). Briefly, blood was initially tested by the Capillus method (Capillus HIV-1/HIV-2 (CP) (Trinity Biotech, Galway, Ireland). Negative results were reported as negative, and no other confirmatory test was done. For positive results a confirmatory test using Serocard (Trinity Biotech, Galway, Ireland) was performed. When results from Serocard were positive, they were reported as positive. However, if the results from Serocard were negative, a tiebreaker test using the Multispot test (Bio-Rad Laboratories, Hercules, CA, USA) was applied, and the results reported as negative or positive depending on the results of this test. All tests were done according to the manufacturer's instructions. HIV was assessed as positive (exposed) or negative (not exposed). These test algorithms have 100% sensitivity and specificity [15]. CD4 count was done using flow cytometry with the Becton Dickinson FACSCount automated instrument (BD FACSCount™, BD Biosciences, Franklin Lakes, NJ, USA).

### HPV testing

The frozen ICC samples and the exfoliated cervical cell samples (in ThinPrep) from control women were shipped to DDL Diagnostic Laboratory (DDL), Voorburg, the Netherlands, for HPV analysis. Frozen samples were shipped in dry ice, while ThinPrep samples were shipped at room temperature. DNA was extracted from the frozen biopsy sections using proteinase K. From the suspension of cervical cells, DNA was isolated by use of a MagNA Pure LC instrument (Roche Diagnostics, Indianapolis, IN, USA) using the total nucleic acid isolation kit (Roche Diagnostics).

SPF_10 _PCR was performed using 10 μl isolated DNA in a final reaction volume of 50 μl. Ten μl of the amplified PCR product was tested with a cocktail of general probes recognising at least 54 mucosal HPV types in a microtitre plate format for the detection of HPV DNA by use of DNA enzyme immunoassay (DEIA). Optical densities (OD_450_) were read on a microtitre plate reader. Subsequently, for HPV DNA-positive samples, 10 μl of the same SPF_10 _amplimer was genotyped by the SPF_10_-LiPA_25 _test (version 1, produced at Labo Biomedical Products, Rijswijk, the Netherlands). By reverse hybridisation of the SPF_10 _amplimer with 28 different probes on the LiPA strip, 25 HPV genotypes can be identified: HPV6, 11, 16, 18, 31, 33, 34, 35, 39, 40, 42, 43, 44, 45, 51, 52, 53, 54, 56, 58, 59, 66, 68/73, 70, 74. The sequence variation within the SPF_10 _primers allows the recognition of these different HPV genotypes, except for HPV68 and 73, as their innerprimer regions are identical and cannot be distinguished in this test. The positive hybridisation on the strips is visualised as a purple band by means of a precipitating colour substrate on the probe site. Samples that were HPV DNA-positive by SPF_10 _PCR-DEIA, but for which no type was identified by the LiPA_25 _strip, were designated as HPVX.

For HPV-negative ICC cases, a beta-globin PCR was performed to assess the quality of DNA isolated from the biopsy.

### Ethical consideration

The study protocol was approved by the Higher Degree's Research Committee of the Faculty of Medicine, Makerere University, and the Uganda National Council of Science and Technology. The study also complied with the ethical norms of Mulago Hospital and those of the Ministry of Health of Uganda. All the study participants signed a written informed consent form before inclusion in the study.

### Data analysis

Our analytical strategy was based on the assumption that normal cervical cells persistently infected with high-risk HPV types may undergo several mutations, which can eventually lead to the development of ICC. In our study cervical samples for HPV testing were collected at the time of cancer diagnosis (and corresponding calendar time among control women). Therefore HPV infections are only to be considered as indicators of persistent HPV infection(s).

We also wanted to test the hypothesis that HIV infection *per se *is associated with cervical cancer risk (as this is a question mark in the literature), and moreover whether the lowered immunity caused by HIV infection or by other causes (as reflected by decreased CD4 count) is associated with this risk. HIV infection and decline in CD4 count may increase the probability of acquiring an HPV infection, or decrease the probability of clearing an HPV infection.

To evaluate the risk of ICC, SCC and adenocarcinoma were analysed separately, and odds ratios (OR) and corresponding two-sided 95% confidence intervals (CI) were calculated using logistic regression models. To address the problem of small sample sizes, we calculated the logistic regression using Firth's penalised likelihood approach [16]. As a consequence of the small sample size it was only possible to estimate the lower or upper limit of the CI for some ORs. We fitted age-adjusted models for SCC and adenocarcinoma of the cervix for women classified according to:

a) Each HPV type or group of HPV types,

b) HIV status (positive or negative),

c) CD4 count per 100 cell decrease, as a linear continuous variable(s),

d) Each HPV type and group of HPV types in subgroups of women classified according to HIV status,

e) HIV status and CD4 count (with groups of >300 or ≤300 and >500 or ≤500 cells) among women who were infected with high-risk HPV types only.

The groups of HPV types analysed were "any HPV infection"; "single HPV infections" for any HPV infection except HPVX; "multiple HPV infections", i.e. concomitant infection with more than one HPV type, except for HPVX; "high-risk HPV types" for infections with any of the HPV types 16, 18, 31, 33, 35, 39, 45, 51, 52, 56, 58, 59, 68 or 73; "low-risk HPV types" for infections with any of the HPV types 6, 11, 34, 40, 42, 43, 44, 53, 54, 66, 70 or 74; "HPV16-related types" for any of the HPV types 16, 31, 33, 35, 52 or 58; "HPV18-related types" for any of the types 18, 39, 45, 59 or 68; and HPVX.

In the analysis of ICC risk according to HIV status, adjustment for CD4 count was done to rule out the possibility that HIV had an influence on risk before affecting immune status (reflected by a decrease in CD4 count).

The statistical software SAS version 9.2 procedure logistic was used for all analyses. All tests of statistical hypotheses were made on the two-sided 5% level of significance.

## Results

During the study period, 484 women with a diagnosis of preliminary incident ICC and 385 control women were approached and invited to participate in the study. Sixty-six (66) potential ICC cases and 22 control women declined to participate, and 41 ICC cases and 44 control women were excluded due to incomplete questionnaires or inability to provide biological samples. Further, 61 potential ICC cases were excluded because ICC diagnosis was not confirmed by histology, as well as five control women who had cervical lesions compatible with cervical intraepithelial neoplasia (CIN). Thus, a total of 316 ICC cases (265 SCC, 42 adenocarcinomas, 5 mixed histology and 4 other histology) and 314 control women were recruited into the study. Of these, cervical samples for HPV testing were obtained from 255 ICC cases (215 SCC, 32 adenocarcinomas and 8 other histological types) and 309 controls. Following HPV testing, 16 of the 255 ICC cases had to be excluded as their samples were beta-globin-negative, leaving 239 ICC cases in the final analysis (Table 1).

**Table 1:** Subject enrolment and measurements

	Cases	%	Controls	%
Women invited to participate	484	100.0	385	100.0
Declined to participate	66	13.6	22	5.7
Incomplete questionnaires	41	8.5	44	11.4
Diagnosis not confirmed^a^	61	12.6	5	1.3
Total preliminarily recruited^b^	316	100.0	314	100.0
Samples for HPV testing^b^	239^c^	75.6	309	98.4
Samples for HIV testing^b^	281	88.9	308	98.1
Combined HIV and HPV samples^b^	222	70.3	303	96.5

Invasive cervical carcinoma study in Kampala, Uganda

^a ^Diagnosis of invasive cervical carcinoma not confirmed by histology for cases; control women found with abnormal cervical smears (cervical intraepithelial neoplasia, not invasive cervical carcinoma).

^b ^Percent based on total recruited.

^c ^In addition to these 239, 16 cases were beta-globin-negative and therefore excluded from all analyses.

Abbreviations: HPV: human papillomavirus.

The clinical and demographic characteristics of study participants are shown in Table 2. A smaller proportion of ICC cases than control women had ever had a Pap smear, and ICC cases had proportionally more HPV infections than control women.

**Table 2:** Clinical and demographic characteristics of invasive cervical carcinoma cases and control women

	Cases^a^		Controls	
	N	% ^b^	N	% ^b^
Number of women	316		314	
Mean age, years (SD)	47	11.5	41	12.5
Mean age at first intercourse, years (SD)	16	3.0	16	3.0
Mean parity (SD)	6.9	5.8	4.8	4.0
Mean CD4 count (SD)	722	375	843	389
Ever used contraceptives	103	32.6	111	35.4
HIV status (+)^c^	55	18.8	54	17.5
Had attended school	207	65.5	271	86.3
Ever smoked	26	8.2	26	8.3
Partner is a current smoker	76	24.1	67	21.3
Alcohol consumption	131	41.5	133	42.4
History of sexually transmitted diseases	148	46.8	137	43.6
Ever had Pap smear	6	1.9	13	4.1
HPV tested	239	75.6	309	98.4
HPV detected	222	92.9^d^	95	30.7^d^
HPV not detected	17^e^	7.1^d^	214	69.3^d^

Invasive cervical carcinoma study in Kampala, Uganda

^a ^Number of women with: squamous cell carcinoma: 265, adenocarcinoma: 42, mixed histology: 5, other: 4.

^b ^Percent where applicable.

^c ^HIV positivity based on available data for 293 cases and 308 controls.

^d ^Percentage (%) of tested.

^e ^Additional 16 invasive cervical carcinoma patients had available samples, but these were beta-globin-negative, indicating poor sample quality, and therefore were excluded from all analyses.

Abbreviations: SD: standard deviation, HPV: human papillomavirus.

Among the 239 ICC cases and 309 control women, HPV DNA was detected in 222 (92.9% of 239) and 95 (30.7% of 309) women, respectively. Seventeen ICC cases (7.1%) were HPV-negative.

There were no other statistically significant differences between ICC cases and control women regarding characteristics, including HIV positivity. The HIV test was positive in 55 (18.8%) ICC cases and 54 (17.5%) control women (Table 2).

### SCC and HPV infection

For SCC, the age-adjusted ORs were statistically significantly increased for any HPV infection (OR 50.8, 95% CI: 25.8-113.9), single HPV infections (OR 33.3, 95% CI: 20.2-56.7), HPV16-related types (OR 11.6, 95% CI: 7.5-18.5), HPV18-related types (OR 9.4, 95% CI: 5.3-17.4), and high-risk HPV types (OR 64.5, 95% CI: 35.4-126.6), in particular HPV16 (OR 29.1, 95% CI: 15.4-60.7), HPV18 (OR 9.6, 95% CI: 4.6-22.3), and HPV45 (OR 58.7, 95% CI: lower limit >7.9).

Multiple HPV infections and infections with low-risk HPV types were not associated with an increased OR for SCC. Adjustment for HIV as well as decline in CD4 count (-100 cells) did not substantially change the OR estimates among the different groups of HPV types (Table 3).

**Table 3:** Risk^a ^of squamous cell carcinoma of the cervix for different human papillomavirus (HPV) types

HPV type	HPV-positive		Age adjusted N = 203 cases & 309 controls		Age and HIV adjusted N = 193 cases & 303 controls		Age, HIV and CD4 adjusted N = 192 cases & 296 controls	
	
	Cases	Controls	OR	95% CI	OR	95% CI	OR	95% CI
High-risk HPV								

All^b^	183	54	64.5	35.4-126.6	84.7	43.5-181.6	82.3	42.2-176.8
16	101	10	29.1	15.4-60.7	28.8	15.2-60.2	27.2	14.4-56.9
18	39	8	9.6	4.6-22.3	8.9	4.2-20.6	8.3	4.0-19.2
31	2	3	0.8	0.1-4.6	0.7	0.1-3.7	0.5	0.1-2.8
33	4	5	1.3	0.3-4.7	1.2	0.3-4.4	1.4	0.3-5.6
35	10	7	2.4	0.9-6.7	2.5	0.9-6.9	2.7	1.0-8.1
39	3	1	3.7	0.6-38.9	4.2	0.7-44.4	4.2	0.7-44.2
45	18	0	58.7	>7.9^j^	56.5	>7.6^j^	57.2	>7.7^j^
51	2	8	0.8	0.2-2.7	0.5	0.1-2.1	0.5	0.1-2.1
52	6	13	0.7	0.2-1.7	0.6	0.2-1.5	0.5	0.2-1.3
56	2	4	0.9	0.2-4.6	1.0	0.2-4.7	1.1	0.2-5.5
58	2	3	0.9	0.2-5.1	1.0	0.2-5.8	1.1	0.2-5.8
59	1	2	0.7	0.1-6.5	0.8	0.1-7.4	0.8	0.1-7.4
68/73	6	7	1.5	0.5-4.6	1.3	0.4-4.0	1.3	0.4-3.9

Low-risk HPV								

All^c^	5	24	0.5	0.2-1.0	0.3	0.1-0.8	0.3	0.1-0.7
6	1	3	1.0	0.1-7.0	1.1	0.1-8.0	1.1	0.1-7.9
11	0	2	0.5	<6.4^k^	0.4	<5.4^k^	0.3	<4.3^k^
34	0	1	0.6	<11.1^k^	0.4	<7.5^k^	0.3	<5.3^k^
40	0	1	0.7	<13.0^k^	0.8	<14.7^k^	0.8	<14.6^k^
42	1	0	4.1	>0.2^j^	^l^	^l^	^l^	^l^
44	0	4	0.2	<2.2^k^	0.3	<2.5^k^	0.2	<2.5^k^
53	0	4	0.1	<1.2^k^	0.1	<1.2^k^	0.1	<1.1^k^
54	1	3	0.4	<2.5^k^	0.4	<2.8^k^	0.5	<2.9^k^
66	0	5	0.4	<2.2^k^	0.1	<1.0^k^	0.1	<1.1^k^
70	1	3	0.7	0.1-4.2	0.6	0.1-3.7	0.5	0.1-3.2
74	2	4	1.0	0.1-4.2	0.9	0.2-4.6	0.8	0.1-3.8

Single inf.^d^	168	49	33.3	20.2-56.7	31.5	19.1-54.0	31.0	18.7-53.5
Multiple inf.^e^	15	20	1.3	0.7-2.7	1.2	0.6-2.4	1.1	0.5-2.3
16-related^f^	123	36	11.6	7.5-18.5	11.9	7.6-19.0	11.4	7.2-18.3
18-related^g^	66	16	9.4	5.3-17.4	8.9	5.0-16.5	8.6	4.9-16.1
HPVX^h^	3	26	0.2	0.1-0.5	0.2	0.1-0.5	0.2	0.1-0.6
Any HPV^i^	186	95	50.8	25.8-113.9	55.7	27.2-132.4	54.9	26.7-130.4

^a ^OR and 95% CI.

^b ^High-risk HPV: 16, 18, 31, 33, 35, 45, 51, 52, 56, 58, 59, 68/73.

^c ^Low-risk HPV: 6, 11, 34, 40, 42, 43, 44, 53, 54, 66, 70, 74.

^d ^Single infection: infection with any of the HPV types above, except HPVX.

^e ^Multiple infections: infections with at least two different HPV types, except HPVX.

^f ^HPV16-related: includes HPV16, 31, 33, 35, 52 and 58.

^g ^HPV18-related: includes HPV18, 45, 59 and 68.

^h ^HPVX: Samples which could not be typed with any of the 25 genotypes above.

^i ^Any HPV infection: infection with any of the HPV types above.

^j ^Indicate only lower limit value of 95% CI.

^k ^Indicate only upper limit value of 95% CI.

^l ^Could not be estimated due to lack of cases with known HIV status.

Abbreviations: inf: infections, OR: odds ratio, CI: confidence interval.

### SCC according to HPV type in HIV-negative and -positive women

The association between infection with different HPV types and SCC was rather similar in HIV-negative and -positive women. However, the number of HIV-positive SCC in our study (N = 42) was limited, and therefore the CIs of our OR estimates were rather broad (Table 4).

**Table 4:** Risk of squamous cell carcinoma of the cervix according to different human papillomavirus (HPV) types and HIV serology

	HIV-negative women						HIV-positive women					
	
HPV types	Cases (N = 162)		Controls (N = 251)				Cases (N = 42)		Controls (N = 52)			
	
	HPV		HPV				HPV		HPV			
	+	-	+	-	**OR**^**a**^	95% CI	+	-	+	-	**OR**^**a**^	95% CI
High-risk^b^	142	10	35	216	76.4	38.8-165.6	41	1	19	33	132.3	>17.0^j^
16	78	74	7	244	32.8	15.6-78.9	23	18	3	49	16.5	5.3-67.6
18	27	125	2	249	22.3	7.1-111.8	12	29	6	46	2.9	1.0-9.1
31	0	152	2	249	0.1	<1.5^i^	2	39	1	51	2.3	0.3-26.4
33	4	148	2	249	2.5	0.5-14.8	0	41	3	49	0.2	<2.7^i^
35	10	142	4	247	4.5	1.5-16.0	0	41	3	49	0.2	<2.0^i^
45	13	139	0	251	41.8	>5.4^j^	5	36	0	52	17.1	1.8-2287

Low-risk^c^	3	149	18	233	0.3	0.1-0.9	2	39	6	46	0.5	0.1-2.0

Single inf.^d^	132	20	38	213	34.0	19.4-62.5	36	5	11	41	21.4	7.5-71.0
Multiple inf.^e^	10	142	9	242	2.0	0.8-5.1	5	36	11	41	0.6	0.2-1.9
16-related^f^	98	54	22	229	17.0	10.0-30.1	25	16	14	38	3.8	1.6-9.4
18-related^g^	49	103	9	242	11.9	5.9-26.4	17	24	7	45	4.5	1.7-13.1
Any HPV^h^	145	7	66	185	51.3	24.8-122.6	41	0	29	23	60.0	>7.7^j^

^a ^All ORs are age-adjusted.

^b ^High-risk HPV: 16, 18, 31, 33, 35, 39, 45, 51, 52, 56, 58, 59, 68, 73.

^c ^Low-risk HPV: 6, 11, 34, 40, 42, 43, 44, 53, 54, 66, 70, 74.

^d ^Single infection: infection with any of the HPV types above.

^e ^Multiple infections: infections with at least two different HPV types.

^f ^HPV16-related: includes HPV16, 31, 33, 35, 52 and 58.

^g ^HPV18-related: includes HPV18, 45, 59 and 68.

^h ^Any HPV infection: infection with any of the HPV types above.

^i ^Indicate only upper limit value of 95% CI.

^j ^Indicate only lower limit value of 95% CI.

Abbreviations: inf: infections, OR: odds ratio, CI: confidence interval.

### Adenocarcinoma and HPV infection

The ORs for adenocarcinoma were statistically significantly increased for any HPV infection (OR 12.9, 95% CI: 5.0-41.8), single HPV infections (OR 13.7, 95% CI: 6.0-34.0), infection with HPV16-related types (OR 3.6, 95% CI: 1.5-8.2), HPV18-related types (OR 22.8, 95% CI: 9.3-58.9), and high-risk HPV types, in particular HPV 16 (OR 11.6, 95% CI: 4.3-31.8), 18 (OR 18.5, 95% CI: 6.6-54.4) and 45 (OR 297.4, 95% CI: lower limit >26.4). Adjustment for HIV status as well as decline in CD4 count (-100 cells) did not substantially change the OR for the different groups of HPV types (Table 5).

**Table 5:** Risk of adenocarcinoma of the cervix for different human papillomavirus (HPV) types^a^

HPV type	Number HPV-positive		Age adjusted		Age and HIV adjusted		Age, HIV and CD4 adjusted	
	
	Cases	Controls	OR	95% CI	OR	95% CI	OR	95% CI
High-risk HPV								

All^b^	26	54	27.2	10.4-88.9	40.5	14.1-157.4	40.0	13.9-155.6
16	10	10	11.6	4.3-31.8	14.5	5.2-42.6	13.8	4.9-40.2
18	10	8	18.5	6.6-54.4	26.3	8.3-96.5	24.0	7.7-86.9
31	1	3	2.2	0.2-16.1	2.6	0.2-19.6	2.6	0.2-19.2
33	0	5	1.2	<12.2^j^	1.4	<14.6^j^	1.3	<14.1^j^
35	0	7	0.8	<7.1^j^	0.9	<8.3^j^	0.8	<7.9^j^
39	0	1	5.5	<108.6^j^	4.7	<92.5^j^	4.7	<92.5^j^
45	4	0	297.4	>26.4^k^	248.3	>22.1^k^	247.8	>22.0^k^
51	2	8	3.9	0.7-16.9	4.1	0.7-17.6	4.1	0.7-17.7
52	0	13	0.3	<2.4^j^	0.4	<3.4^j^	0.3	<2.9^j^
56	0	4	0.8	<11.0	1.0	<12.1^j^	1.0	<12.8^j^
58	0	3	1.3	<15.5^j^	1.3	<14.9^j^	1.3	<14.9^j^
59	0	2	0.9	<18.6^j^	1.1	<19.27^j^	1.1	<19.8^j^
68/73	2	7	3.0	0.5-13.7	3.6	0.6-16.2	3.4	0.6-16.0

Low-risk HPV								

All^c^	1	24	0.6	0.1-2.4	0.6	0.1-2.7	0.6	0.1-2.6
6	1	3	12.8	1.1-96.7	10.0	0.9-76.0	10.0	0.9-75.9
11	0	2	3.7	<55.1^j^	4.6	<64.5^j^	3.8	<56.5^j^
34	0	1	4.3	<82.4^j^	6.4	<161^j^	4.6	<126.4^j^
40	0	1	5.3	<102.7^j^	4.5	<88.3^j^	4.5	<88.3^j^
44	1	4	5.4	0.5-35.2	4.8	0.4-30.3	4.8	0.4-30.3
53	0	4	0.7	<7.6^j^	0.8	<8.7^j^	0.8	<8.3^j^
54	0	3	0.6	<7.4^j^	0.7	<8.0^j^	0.7	<8.0^j^
66	0	5	0.9	<9.0^j^	1.2	<12.7^j^	1.0	<11.2^j^
70	0	3	1.3	<15.3^j^	1.6	<17.7^j^	1.4	<16.0^j^
74	0	4	0.8	<11.1^j^	0.9	<11.9^j^	0.9	<11.5^j^

Single inf.^d^	22	49	13.7	6.0-34.0	15.5	6.6-40.5	15.3	6.5-40.0
Multiple inf.^e^	4	20	2.5	0.7-7.5	3.3	0.9-10.5	3.1	0.8-9.8
16-related^f^	11	36	3.6	1.5-8.2	4.9	2.0-11.6	4.7	1.9-11.2
18-related^g^	16	16	22.8	9.3-58.9	26.2	10.3-71.8	24.5	9.7-67.0
HPVX^h^	0	26	0.2	<1.2^b^	0.2	<1.3^b^	0.2	<1.5^b^
Any HPV^i^	26	95	12.9	5.0-41.8	18.7	6.6-71.6	18.6	6.6-71.2

^a ^Analysis conducted with 32 cases and 309 controls.

^b ^High-risk HPV: 16, 18, 31, 33, 35, 39, 45, 51, 52, 56, 58, 59, 68, 73.

^c ^Low-risk HPV: 6, 11, 34, 40, 42, 43, 44, 53, 54, 66, 70, 74.

^d ^Single infection: infection with any of the HPV types above, except HPVX.

^e ^Multiple infections: infections with at least two different HPV types, except HPVX.

^f ^HPV16-related: includes HPV16, 31, 33, 35, 52 and 58.

^g ^HPV18-related: includes HPV18, 45, 59 and 68.

^h ^HPVX: Samples which could not be typed with any of the 25 genotypes above.

^i ^Any HPV infection: infection with any of the HPV types above.

^j ^Indicate only upper limit value of 95% CI.

^k ^Indicate only lower limit value of 95% CI.

Abbreviations: inf: infections, OR: odds ratio, CI: confidence interval.

### Adenocarcinoma according to HPV type in HIV-negative and -positive women

Given the very low number of HIV-positive adenocarcinomas (N = 4) in our study, our risk estimates are not sufficiently precise to reach any conclusions about differences between HIV-negative and -positive women, but there was no evidence of a difference in risk (Table 6).

**Table 6:** Risk of adenocarcinoma of the cervix according to different human papillomavirus (HPV) types and HIV serology

	HIV-negative women						HIV-positive women					
	
HPV types	Cases (N = 28)		Controls (N = 251)				Cases (N = 4)		Controls (N = 52)			
	
	HPV		HPV				HPV		HPV			
	+	-	+	-	**OR**^**a**^	95% CI	+	-	+	-	**OR**^**a**^	95% CI
High-risk^b^	24	3	53	216	39.5	13.6-153.8	2	0	19	33	8.8	0.7-1235
16	8	19	7	244	11.0	3.6-34.2	2	0	3	49	108.2	>4.0^i^
18	10	17	2	249	57.9	14.8-327.8	0	2	6	46	1.3	<20.8^j^
31	1	26	2	249	2.8	0.2-24.2	0	2	1	51	6.0	<149.8^j^
33	0	27	2	249	1.8	<23.7^j^	0	2	3	49	3.5	<71.4^j^
45	4	23	0	251	251.3	>22.2^i^	0	2	0	52	^k^	^k^

Low-risk^c^	1	26	18	233	0.7	0.1-3.2	2	0	6	46	1.4	<19.8^j^

Single inf.^d^	7	20	38	213	14.5	6.0-38.2	2	0	11	41	15.0	1.2-2086
Multiple inf.^e^	4	23	9	242	5.0	1.3-16.9	2	0	11	41	0.9	<11.0^j^
16-related^f^	9	18	22	229	4.3	1.6-10.5	2	0	14	38	12.8	1.0-1794
18-related^g^	16	11	7	244	42.2	15.0-133.8	0	2	7	45	1.3	<17.8^j^
Any HPV^h^	24	3	66	185	18.3	6.4-70.0	2	0	29	23	4.0	0.3-565.6

^a ^All ORs are age-adjusted.

^b ^High-risk HPV: 16, 18, 31, 33, 35, 39, 45, 51, 52, 56, 58, 59, 68, 73.

^c ^Low-risk HPV: 6, 11, 34, 40, 42, 43, 44, 53, 54, 66, 70, 74.

^d ^Single infection: infection with any of the HPV types above.

^e ^Multiple infections: infections with at least two different HPV types.

^f ^HPV16-related: includes HPV16, 31, 33, 35, 52 and 58.

^g ^HPV18-related: includes HPV18, 45, 59 and 68.

^h ^Any HPV infection: infection with any of the HPV types above.

^i ^Indicate only lower limit value of 95% CI.

^j ^Indicate only upper limit value of 95% CI.

^k ^Could not be estimated due to lack of cases with known HIV status.

Abbreviations: inf: infections, OR: odds ratio, CI: confidence interval.

### SCC and adenocarcinoma according to HIV status

Table 7 shows the overall OR for SCC and adenocarcinoma in relation to HIV infection. There was no association between HIV positivity and SCC (OR 1.5, 95% CI: 0.9-2.3) or adenocarcinoma (OR 1.1, 95% CI: 0.4-2.7) in age-adjusted models.

**Table 7:** Odds ratios (OR) and 95% confidence intervals (CI) for squamous cell carcinoma and adenocarcinoma of the cervix according to HIV infection and adjustment factors: age, CD4 count (decrease of 100 cells), and human papillomavirus (HPV) types

	Squamous cell carcinoma **N = 246 cases, 308 controls**^**a**^		Adenocarcinoma **N = 39 cases, 308 controls**^**b**^	
	
Adjustment factor	OR for HIV-positive vs. HIV-negative	95% CI	OR for HIV-positive vs. HIV-negative	95% CI
Age	1.5	0.9-2.3	1.1	0.4-2.7
Age + CD4	1.6	1.0-2.6	0.8	0.1-2.6
Age + CD4 + HPV16	1.6	0.9-2.9	0.5	0.1-1.8
Age + CD4 + HPV16-related^c^	1.4	0.7-2.4	0.5	0.1-1.6
Age + CD4 + HPV18	1.4	0.8-2.4	0.3	<1.2^j^
Age + CD4 + HPV18-related^d^	1.4	0.8-2.4	0.4	0.1-1.5
Age + CD4 + High-risk HPV^e^	0.6	0.3-1.1	0.3	<0.9^j^
Age + CD4 + Low-risk HPV^f^	1.9	1.1-3.2	0.8	0.2-2.6
Age + CD4 + Single HPV inf.^g^	1.2	0.6-2.4	0.5	0.1-1.7
Age + CD4 + Multiple HPV inf.^h^	1.8	1.1-3.0	0.6	0.1-2.0
Age + CD4 + Any HPV inf.^i^	0.8	0.4-1.4	0.4	0.1-1.2

^a ^HIV-positive: 48 cases, 54 controls.

^b ^HIV-positive: 5 cases, 54 controls.

^c ^HPV16-related: includes HPV16, 31, 33, 35, 52 and 58.

^d ^HPV18-related: includes HPV18, 45, 59 and 68.

^e ^High-risk HPV: 16, 18, 31, 33, 35, 39, 45, 51, 52, 56, 58, 59, 68, 73.

^f ^Low-risk HPV: 6, 11, 34, 40, 42, 43, 44, 53, 54, 66, 70, 74.

^g ^Single infection: infection with any of the HPV types above.

^h ^Multiple infections: infections with at least two different HPV types.

^i ^Any HPV infection: infection with any of the HPV types above.

^j ^Indicate only lower limit value of 95% CI.

Abbreviations: inf: infections,.

In statistical models adjusting for age and decline in CD4 count (-100 cells), HIV-positive women had an increased OR for SCC (OR 1.6, 95% CI: 1.0-2.6) of borderline significance. However, the OR for SCC among HIV-positive women was no longer statistically significant after further adjustment for different groups of HPV infections (adjustment for age, CD4 count and any HPV infection: OR 0.8, 95% CI: 0.4-1.4), except when we adjusted for infection with low-risk HPV types alone (OR 1.9, 95% CI: 1.1-3.2) and multiple HPV infections (OR 1.8, 95% CI: 1.1-3.0), when the lower CIs were of borderline statistical significance (Table 7). There was no evidence of association between HIV infection and adenocarcinoma in any of the statistical models tested in Table 7.

### SCC and adenocarcinomas according to CD4 count

Decline in CD4 count (-100 cells) was not statistically significantly associated with SCC (OR 1.2, 95% CI: 1.0-1.4) or adenocarcinoma (OR 0.8, 95% CI: 0.1-2.6) in age-adjusted models including all women with available information in the study, nor in analyses adjusting for HIV and individual HPV types, or groups of HPV types (data not shown).

### HIV infection, CD4 count and SCC and adenocarcinoma risk among women infected with high-risk HPV types

Table 8 shows the overall age-adjusted OR for SCC and adenocarcinoma according to HIV infection and decline in CD4 count (-100 cells) among women who were infected with high-risk HPV types (i.e. ICC cases and control women who were not infected with a high-risk HPV type were excluded from this analysis). HIV positivity was not associated with SCC risk (OR 0.7, 95% CI: 0.3-1.4), but it was associated with decreased adenocarcinoma risk (OR 0.2, 95% CI: 0.04-0.8). However, only two adenocarcinomas were HIV-positive among women infected with high-risk HPV types, and therefore this inverse association may be a chance finding. There was no clear association between CD4 count and SCC or adenocarcinoma.

**Table 8:** Age adjusted odds ratio (OR) and 95% confidence interval (CI) for invasive cervical carcinoma according to HIV serology and CD4 count among women who are infected with high-risk human papillomavirus (HPV) types^a^

	Controls	Squamous cell carcinoma			Adenocarcinoma		
	Number	Number	OR	95% CI	Number	OR	95% CI
HIV							
Any (+or-)	54	183			26		
Negative	35	142	1.0 (ref)		24	1.0 (ref)	
Positive	19	41	0.7	0.3-1.4	2	0.2	0.04-0.8
CD4 count							
Any	37	135			21		
>300	33	113	1.0 (ref)		19	1.0 (ref)	
≤300	4	22	1.8	0.6-6.2	2	1.8	0.3-10.1
>500	26	90	1.0 (ref)		15	1.0 (ref)	
≤500	11	45	1.4	0.6-3.3	6	0.9	0.3-3.1

^a^High-risk HPV: 16, 18, 31, 33, 35, 39, 45, 51, 52, 56, 58, 59, 68, 73.

NOTE: CD4 count classified according to two different cut-points: 300 and 500 cells.

## Discussion

The HPV types associated with both SCC and adenocarcinoma risk in our study have also been found to be associated with most ICC in other regions of the world [17,18].

### SCC and HPV infection

Our study shows that SCC in Uganda is strongly associated with any HPV infection and infection with high-risk HPV types, in particular HPV16 (and related types), 18 (and related types) and 45. We found no evidence that adjustment for HIV infection or CD4 decline affect the OR for HPV infections significantly.

As in our present study, several earlier studies found that HPV16 predominated in SCC [19]. Our study showed higher ORs for SCC associated with HPV16 (OR 29.1, 95% CI: 15.4-60.7), which is similar to a study from Taiwan by Chen et al. [20], where HPV16 was associated with an OR of 67. The Taiwanese study found substantial elevated risks for SCC associated with HPV52 (OR 3.04) and HPV58 (OR 5.22), which were not confirmed in our study, while the SCC risk with the other HPV types were low. A study from Mexico also found a high risk of cervical cancer for HPV16 and 18 [1]. The results from Mexico are exceptional in that the OR for HPV18 was higher than that of HPV16. High risks of cervical cancer for HPV18 have also been reported by a number of authors [21-23]. Low risks for HPV18 have been found in some studies from India [24,25].

### Adenocarcinomas and HPV infection

For adenocarcinoma, we found an association between any HPV infection, in particular single infections, infections with high-risk HPV types, and most importantly HPV16 (and related types), 18 (and related types) and 45. Adjustment for HIV infection and decline in CD4 count did not alter the associations meaningfully, suggesting that there is only a small confounding effect of HIV and its associated immune deficiency on the risk of HPV in cervical adenocarcinoma.

HPV18 has been reported as the most frequent HPV type found in adenocarcinoma [19]. In a pooled analysis of case-control studies, the relative risk of HPV18 in cervical adenocarcinoma was high (OR 410.32, 95% CI: 167.44-∞) [4]. These results were much higher than ours for HPV18 (OR 18.5, 95% CI: 6.6-54.4).

These differences in findings across different studies may indicate that regional factors, such as differences in the prevalence of different HPV types, may affect ICC risk [26]. These differential risks are probably due to the pathogenesis of the two major types of ICC [27]. Smith et al. [28] and Madeleine et al. [29] noted an elevated risk of SCC associated with *Trichomonas vaginalis*, but no associated increased risk for adenocarcinoma. Another factor which could in theory explain the differences in SCC and adenocarcinoma risk is smoking [30]; however smoking is not common in Uganda, with a prevalence of only 3% among adult women [31] and 5.3% among adolescent girls [32]. The relatively low relative risk of ICC due to other high-risk HPV types (besides HPV16, 18 and 45) is supported by the low prevalence of these HPV types in ICC in several other studies [3,33], including a recent study by de Cremoux et al. [34] which found that up to one-third of ICC were not associated with HPV16 or 18. Some of these high-risk HPV types, namely HPV31, 33, 52 and 58, have been found to be more prevalent in high-grade intraepithelial cervical lesions than in SCC [17].

As possible caveats, our study was limited by the number of probes for HPV, which only allowed us to detect 25 HPV genotypes. These are, however, the most important HPV types described in the literature as being associated with cervical lesions including ICC. Moreover, the SPF_10_-LiPA_25 _HPV test used in our study (version 1, produced at Labo Biomedical Products, Rijswijk, the Netherlands) is highly sensitive for the HPV types tested. We tested HPV-negative ICC samples for beta-globin, and excluded from all analyses those women whose samples were beta-globin-negative. Thus, the samples included in our analysis were of sufficient quality to allow HPV detection. However, since cases were in general presenting at advanced stages (3 and 4), it is possible that HPV could no longer be detected in the cervical cells of some patients, which may explain why a small proportion of our cases were HPV-negative. This could have somewhat biased the OR for the associations between HPV and ICC towards unity.

### HIV and CD4 count

Of particular interest was the lack of clear association between HIV infection and decline in CD4 count on the risk for SCC in our entire study population, and in the subset of women infected with high-risk HPV types. Although there was a non-significant increase in SCC risk associated with HIV infection, it is not probable that this effect was due to immune depression on disease progression, since the OR was not decreased when adjusting for CD4 count.

For adenocarcinomas, there was no evidence of association with HIV infection in the entire study population, regardless of adjustment for different HPV types or groups of types and CD4 count. However, there was an inverse association between HIV infection and adenocarcinoma risk when restricting the analysis to women who were infected with high-risk HPV types. There was no association between CD4 count and adenocarcinoma risk when analysing all women in our study, or when restricting the analyses to the subset of women infected with high-risk HPV types. These finding must be interpreted with caution, as the number of HIV-positive women in our study, in particular among adenocarcinoma cases, was small, and therefore we cannot rule out the possibility of a chance finding.

The overall HPV prevalence among HIV-positive control women was higher than among HIV-negative control women. This may be explained by common sexual transmission routes, or it may be a reflection of the HIV-associated decrease in immune response, which increases the probability of being infected with HPV, and of clearing the infection less effectively than women who are not infected with HIV. As a consequence, the association between HPV infection and ICC among HIV-positive women was in general weaker than among HIV-negative women.

Several previous studies have shown varying risk of ICC in association with HIV infection [12,35-41]. Earlier studies suggested that a possible increase in cervical cancer risk among HIV-positive women could be attributed to the increased prevalence of HPV infection, or persistence of HPV infection [42,43]. In addition, CIN is less likely to regress, and therefore the probability of CIN progressing to ICC is increased in HIV-infected women [43]. This has been attributed to reduced HLA class II molecules and the presence of immature Langerhans cells within the cervical tissue in HIV-infected women [44]. Other studies have suggested that HPV types other than 16 or 18 are more prevalent in HIV-positive women [19,45], and that the median duration of infections with different individual HPV types lasts longer in HIV-positive women as compared to HIV-negative women, with the exception of HPV16, which seem to be similar [46,47]. This indicates that the probability of progression of cervical pre-invasive lesions to invasive lesions may be increased among HIV-positive women.

In our case-control study design, cases were recruited when ICC was already present, and exposures were measured at the time subjects were recruited into the study. Thus, we do not know if HIV infection, or if decrease in CD4 count, occurred before or after the onset of HPV infection or cervical lesions. It is possible that some HIV infections occurred before the onset of HPV infections and before the development cervical pre-invasive or invasive lesions. HIV infection could also have occurred while women were infected with HPV, thus eventually decreasing their capacity to clear the infection. It is also plausible that HIV infections may have occurred too late in life to have any effect on the occurrence or persistence of HPV infection, as suggested by a study from Nairobi, Kenya [48].

The lack of association between HIV infection, CD4 count and SCC risk in our study could also be explained by the fact that progression of CIN to ICC is multifactorial, and not dependent on immune status only [49], by the existence of slow progressors [50,51] or elite controllers [52,53] in some women, or by the possibility that HIV and CD4 count have no role in the progression of cervical cancer.

Our study had a number of advantages compared to previous studies. We enrolled all consecutive patients suspected to have ICC, aiming to reduce bias that could occur if any criteria associated with the exposure were used to select ICC cases, for example stage, duration of symptoms, tribe, etc. While other studies have used women with other disease conditions as controls [11,12], we were able to use women who were not hospitalised, had no cervical disease (CIN cases were excluded), and were living in the same geographical areas as ICC cases. Further, they were representative of the population base from where the cases arose. Since the evaluation of family history of cervical cancer was not part of our study hypothesis, the inclusion of blood relatives of the ICC cases as controls did not constitute a problem.

Another strength was that we carried out the study in an area where both HIV prevalence [14] and cervical cancer incidence are high [13]. This allowed us to adjust for HIV status and decline in CD4 count in our HPV analysis, as well as to perform stratified analyses by HIV status to assess the impact of HIV infection *per se *and the HPV risk associated with HIV status on ICC.

Our study had a relatively small number of HIV-positive SCC and, in particular, adenocarcinoma. In addition, assessment of immunity was based only on CD4 count, which may reflect HIV infection on the systemic immune system. Although CD4 count has been used before [54], it is difficult to know when immunodeficiency sets in. The statistical power for detecting cancer risk in subgroups of HIV-infected women was limited, in particular for adenocarcinoma.

## Conclusions

In conclusion, the OR for SCC and adenocarcinoma of the cervix in Uganda were increased in women infected with HPV, in particular single HPV infections, infections with HPV16- and 18-related types and infections with high-risk HPV types, specifically with HPV16, 18 and 45. HIV infection and CD4 count were not clearly associated with SCC or adenocarcinoma in our entire study population. Among women infected with high-risk HPV types, there was no association between SCC, HIV and CD4 count, while there was an inverse association between HIV infection - but not CD4 count - and adenocarcinoma.

## List of abbreviations

AIDS: acquired immune deficiency syndrome; BD FACSCount: automated instrument for monitoring of HIV/AIDS; CD4: cluster of differentiation 4, glycoprotein, in humans encoded by the *CD4 *gene; CI: confidence intervals; CIN: cervical intraepithelial neoplasia; DDL: DDL Diagnostic Laboratory; DEIA: DNA enzyme immunoassay; DNA: deoxyribonucleic acid; HIV: human immunodeficiency virus; HPV: human papillomavirus; ICC: invasive cervical carcinoma; OR: odds ratio; PCR: polymerase chain reaction; SCC: squamous cell carcinoma. SPF_10_-LiPA_25_: system for HPV genotyping

## Competing interests

The authors declare that they have no competing interests.

## Authors' contributions

MO, FM and EW were responsible for developing the concept, full proposal development and obtaining ethical approvals. MO carried out the field work and was responsible for data collection. BK and WQ made all laboratory analyses and contributed to the interpretation of laboratory results. SS provided statistical analyses and interpretation of the results. MO and EW wrote the manuscript while all other authors; FM, BK, WK and SS gave their critical comments upon the writing process and revised the final manuscript. All authors approved the version to be published.
